# Comparing the Health State Preferences of Older Persons, Informal Caregivers and Healthcare Professionals: A Vignette Study

**DOI:** 10.1371/journal.pone.0119197

**Published:** 2015-03-04

**Authors:** Cynthia S. Hofman, Peter Makai, Jeanet W. Blom, Han Boter, Bianca M. Buurman, Marcel G. M. Olde Rikkert, Rogier Donders, René J. F. Melis

**Affiliations:** 1 Radboud university medical center, Department of Geriatric Medicine (HP 925), Nijmegen, The Netherlands; 2 Radboud university medical center, Department for Health Evidence (HP133), Nijmegen, The Netherlands; 3 Leiden University Medical Centre, Department of Public Health and Primary Care, Leiden, The Netherlands; 4 University of Groningen, University Medical Centre Groningen, Department of Epidemiology (FA41), Groningen, The Netherlands; 5 Academic Medical Center, Department of Internal Medicine, section of Geriatric Medicine (F4–108), Amsterdam, The Netherlands; Iranian Institute for Health Sciences Research, ACECR, IRAN, ISLAMIC REPUBLIC OF

## Abstract

**Background:**

The Older Persons and Informal Caregivers Survey—Minimum Dataset (TOPICS-MDS) collects uniform information from research projects funded under the Dutch National Care for the Elderly Programme. To compare the effectiveness of these projects a preference-weighted outcome measure that combined multidimensional TOPICS-MDS outcomes into a composite endpoint (TOPICS-CEP) was developed based on the health state preferences of older persons and informal caregivers.

**Objectives:**

To derive preference weights for TOPICS-CEP’s components based on health state preferences of healthcare professionals and to investigate whether these weights differ between disciplines and differ from those of older persons and informal caregivers.

**Materials and Methods:**

Vignette studies were conducted. Participants assessed the general wellbeing of older persons described in vignettes on a scale (0-10). Mixed linear analyses were used to obtain and compare the preference weights of the eight TOPICS-CEP components: morbidities, functional limitations, emotional wellbeing, pain experience, cognitive problems, social functioning, self-perceived health, and self-perceived quality of life (QOL).

**Results:**

Overall, 330 healthcare professionals, 124 older persons and 76 informal caregivers participated. The preference weights were not significantly different between disciplines. However, the professionals’ preference weights differed significantly from those of older persons and informal caregivers. Morbidities and functional limitations were given more weight by older persons and informal caregivers than by healthcare professionals [difference between preference weights: 0.12 and 0.07] while the opposite was true for pain experience, social functioning, and self-perceived QOL [difference between preference weights: 0.13, 0.15 and 0.26].

**Conclusion:**

It is important to recognize the discrepancies between the health state preferences of various stakeholders to (1) correctly interpret results when studying the effectiveness of interventions in elderly care and (2) establish appropriate healthcare policies. Furthermore, we should strive to include older persons in our decision making process through a shared decision making approach.

## Background

The population is aging across the world. This demographic shift will lead to extraordinary demands on our healthcare system [1]. With the limited financial resources and insufficient number of healthcare professionals, evaluating the effectiveness of healthcare interventions has become an integral part of health policy and decision-making [2]. However, it is a great challenge to evaluate interventions for elderly because their health states are complex and interventions often target more than one domain [3].

An expert panel of The American Geriatrics Society formulated guiding principles on how clinicians should approach the care of older adults with multi-morbidity. Several steps were defined including “Consider patient preferences” and “Is relevant evidence available regarding *important* outcomes?”. These principles are also crucial for researchers evaluating the effectiveness of intervention in older adults [4]. Hence, a generic measurement instrument with a composite endpoint (CEP) that is preference based and includes important outcomes would be helpful to compare outcomes across groups, thereupon, to establish and compare the effectiveness of different geriatric interventions [5, 6].

The Dutch National Care for the Elderly Programme (NCEP) was established in 2008 to promote proactive, integrated healthcare for older persons with complex healthcare needs [7]. Within the NCEP The Older Persons and Informal Caregivers Survey Minimum DataSet (TOPICS-MDS) was developed to collect uniform information from all research project funded under this Programme. A detailed description of TOPICS-MDS has been presented elsewhere [8]. Briefly, TOPICS-MDS is a collection of four validated instruments which was designed to collect essential information on the physical and mental wellbeing of older persons [9] and informal caregivers [10] in the Netherlands. The survey was administered in multiple research settings to elicit uniform outcome data in the aim of creating a national data repository on older persons’ health. Over 60 NCEP research projects have already incorporated TOPICS-MDS in their research protocol and evaluated more than 32,000 participating elderly using the survey [8].

To compare the effectiveness of these projects a preference-weighted outcome measure that combined multidimensional TOPICS-MDS outcomes into a composite endpoint (TOPICS-CEP) was developed based on the health state preferences of older persons and informal caregivers [11]. The benefit of using TOPICS-CEP is that the overall value of interventions can be calculated in a standardized manner which makes the evaluation process easier and more objective.

Briefly, TOPICS-CEP is a preference-weighted index ranging from 0 (worst possible general wellbeing) to 10 (best possible general wellbeing). It combines 42 data points from TOPICS-MDS covered by eight components, such as functional limitations (Katz index of independence)[12] and emotional wellbeing (mental health subscale of the RAND-36) [13]. The components vary in both scale range and preference weight. Raw TOPICS-CEP scores are transformed into indexed scores. More detailed information about the development of TOPICS-CEP and its scoring procedure can be found elsewhere [11, 14].

The various stakeholders in geriatrics share a mutual goal which is to improve a person’s health and wellbeing. However, studies have shown significant differences between the perspectives of older persons and their healthcare professionals [15, 16]. Consequently, we expected that the preference weights of the TOPICS-CEP’s components would differ between those of older persons and their healthcare professionals, which could potentially lead to treatment decisions by professionals that are at odds with patient preferences and to incorrect interpretation of findings in effectiveness studies. For that reason, we explored the TOPICS-CEP components’ weights based on the health state preferences of healthcare professionals in this current study and compared them with the weights based on the health state preferences of older persons and informal caregivers found in our previous study.

In short, the primary objectives of this study were: (1) to examine the association of preference weights with the healthcare professionals’ characteristics; (2) to examine the difference between healthcare professionals’ preference weights and those of older persons and informal caregivers; and (3) to explore the influence of the cases’ gender and age on the distribution of the composite scores.

## Method

### Ethical approval

The Medical Ethics Committee of the Radboud University Medical Center formally stated that this study was exempt from ethical review (Radboud University Medical Center Ethical Committee review reference number: CMO: 2010/244). Written informed consent was obtained from the older persons and informal caregivers who participated in our previous study.

### Study design

Vignette studies were conducted to obtain the preference weights for the eight TOPICS-CEP components: morbidities (list of 17 pre-defined conditions) [17], functional limitations (Katz index of independence) [12], emotional wellbeing (mental health subscale of the RAND-36) [13], pain experience (pain dimension of the EQ-5D) [18], cognitive problems (cognition dimension of the EQ-5D+C] [18], social functioning (item 10 from the RAND-36) [13], self-perceived health (item 1 from the RAND-36) [13], self-perceive QOL (phrasing similar to self-perceived health item from the RAND-36) [13]. The participants rated the general wellbeing (GWB) of case vignettes, which were short descriptions or profiles of older persons (further called: cases).

### Participants

**First vignette study: Older persons and informal caregivers**. In the first vignette study, 124 community dwelling older persons and 76 informal caregivers participated as raters. They were recruited and their data was collected by four academic centres: Radboud University Medical Center, University Medical Centre Groningen, Academic Medical Centre, and Leiden University Medical Centre. A full report and more detailed information can be found elsewhere [11].

**Second vignette study: Healthcare professionals**. The 330 healthcare professionals who rated the cases in the second vignette study were recruited during two national geriatric conferences in February 2012 and October 2012, via websites of various professional associations, and via the website of NCEP. The professionals worked as physicians, nurses, welfare staff or allied health professional across the Netherlands, covering both urban and more rural parts of the country.

### Material

The vignettes were based on data of a sample of cases derived from TOPICS-MDS data repository, which consists of pooled data from various research projects which differ across study design, sampling framework, and inclusion criteria. In general, each vignette included 46 items covering the eight previously described TOPICS-CEP components: morbidities, functional limitations, emotional wellbeing, pain experience, cognitive problems, social functioning, self-perceived health, and self-perceive QOL. The information included in the vignettes regards all the variables (or items) from TOPICS-MDS for older persons which carry information relevant for understanding an individual’s outcome. This excludes demographics and health service utilization. Excluding these components was based on the rationale that demographics such as gender and age and health service utilization cannot be influenced by healthcare delivery.

By using empirical data, vignettes with plausible health state combinations were constructed. We made sure that the complete ranges of outcomes for the different health domains were covered. All raters evaluated a limited number of cases and we assured that all cases were rated by a sufficiently large number of raters. Since it was to be expected that some of the disciplines would consist of lower numbers of participants, we used a smaller set of cases in this present study. To guarantee that each discipline evaluated the complete range of the outcomes we chose a new set of cases for this study.

**First vignette study: Older persons and informal caregivers**. The cases (N = 292) of whom the GWB were assessed by older persons and informal caregivers had a mean age (±SD) of 81.4 (5.72) years and 58.6% (N = 171) was female. The majority of these cases were either married (42.8%, N = 125) or their partner was deceased (42.8%, N = 125), and 39.7% (N = 116) lived independently with someone, e.g. a partner or family member.

**Second vignette study: Healthcare professionals**. The cases (N = 161) of whom the GWB were assessed by healthcare professionals had a mean age (±SD) of 82.4 (6.5) years and 67.7% (N = 109) was female. The majority of these cases were either married (28.0%, N = 45) or their partner was deceased (57.1%, N = 92), and 43.5% (N = 70) lived in either a nursing home or a residential care facility. An overview of the health domains, items per domain, and levels per item which were included in the vignettes and used in the statistical analyses can be found in S1 Appendix.

### Procedure

After reading each vignette (an example can be found in S2 Appendix), raters were asked to give a score ranging from zero to ten representing how bad or good, in their opinion, the GWB of the described case was.

**First vignette study: Older persons and informal caregivers**. The vignette study within the group of older persons and informal caregivers was conducted on paper. After two trial cases, which were the same for every participant within the study, the raters were asked to give scores to a random selection of ten cases. More information about the exact procedure can be found elsewhere [11].

**Second vignette study: Healthcare professionals**. The healthcare professionals had the opportunity to evaluate the cases on paper or online via the website of QuestionPro (online survey software to create, publish, and distribute online surveys); both the hardcopy and the online survey had the same format and the participants had to follow the same procedure. After a trial case, which was the same for every participant, the raters were asked to give scores to a random selection of five cases. In addition, we asked them to answer a couple of questions regarding: age, gender, occupation, number of years in this occupation, and number of patients/clients aged ≥ 65 years per week.

### Statistical analysis

The statistical procedures for both vignette studies were comparable. The analyses to derive the preference weights for TOPICS-CEP’s components based on the health state preferences of older persons and informal caregivers can be found elsewhere [11]. To derive the weights for the components based on the health state preferences of the healthcare professionals five mixed linear regression models were constructed. Each model had the following structure: (1) The GWB scores were used as dependent variable; (2) The eight CEP components were used as independent variables (predictors): morbidities, functional limitations, emotional wellbeing, pain experience, cognitive functioning, social functioning, self-perceived health, and self-perceived QOL; and (3) To correct for clustering within raters a random (rater dependent) intercept was included. Furthermore, we included in each model one of the following five factors: *profession* (physician, nurse, welfare staff, and allied health professional), *physicians’ discipline* (general practitioner, nursing home physician, internist, geriatrician), *years of experience*, *number of patients aged ≥65 years per week*, *or rater group* (healthcare professional / older person or Informal caregiver) together with the interaction between the included factor and each of the CEP components. The parameter estimates for the eight domains represent the preference weights.

Subsequently, for the cases used in both vignette studies, we described the distribution of TOPICS-CEP scores (based on the preference weights of older persons and informal caregivers) across cases’ gender and age groups and compared them with the distribution of such a composite score when one would base it on healthcare professionals’ preferences (further referred to as: HP’s CEP). A paired sample T-test was used to examine the difference between TOPICS-CEP and the HP’s CEP. In addition, to explore the level of agreement between the two composite outcome measures a Bland-Altman plot was used.

## Results

The healthcare professionals who participated as raters in this study had a mean age of 43.0 years (SD 11.0) and 80.3% was female (N = 265). The majority of the healthcare professionals conducted the vignette experiment online (76.7%, N = 253). Additional information about the characteristics of the healthcare professionals can be found in Table 1.

**Table 1:** Distribution of the healthcare professionals (N = 330).

			Years active in current profession		Number of patients / clients ≥ 65 years per week	
	N	%	Mean	SD	Mean	SD
Physicians	127	38.5	10.7	8.5	30.5	22.2
Nurses	102	30.9	13.0	10.6	21.1	15.7
Welfare staff	45	13.6	10.6	9.2	14.0	14.3
Allied health professionals	56	17.0	12.7	9.8	21.9	14.7
Total	330	100	11.8	9.5	23.9	19.1

In our previous study, older persons (N = 124) and informal caregivers (N = 76) participated as raters. The older persons had a mean age of 78.3 years (SD 6.7) and 62.9% (N = 78) was female. The informal caregivers had a mean age of 62.9 years (SD 12.1) and 72.4% (N = 55) was female.

### Healthcare professionals’ characteristics and their preference weights

The models including the interaction terms between *profession*, *physician’s discipline*, or *number of patients aged 65 years and older* with each of the predictors showed no significant interaction effects. In contrast, the model that included the interaction terms between *years of experience* and each of the predictors showed a significant interaction effect between years of experience and *morbidities* (p = 0.02). For each additional year of experience, the preference weight of the component *morbidities* declined with 0.01 points. Hence, the association between the number of morbidities and GWB score became less strong.

### Comparing the preference weights of healthcare professionals with those of older persons and informal caregivers

For several components of TOPICS-CEP the healthcare professionals’ preference weights differed significantly from those of older persons and informal caregivers. The components’ weights based on the health state preferences of older persons and informal caregivers versus those based on the preferences of healthcare professionals can be found in Table 2. Significant interaction effects were found between the factor *healthcare professional* and the outcome domains: morbidities, functional limitations, pain experience, social functioning, and self-perceived QOL (p<0.05). The estimated differences of these preference weights were: -0.12, -0.07, 0.14, 0.15 and 0.26, respectively. These estimates weights indicated that morbidities and functional limitations were given more weight by older persons and informal caregivers than by healthcare professionals, whereas the opposite was true for pain experience, social functioning, and self-perceived QOL.

**Table 2:** Mixed linear regression model: Unstandardized coefficients with corresponding p-values and confidence interval, standardized coefficients, and p-value for the interaction effect between each predictor and the independent variable healthcare provider.

	Healthcare Professionals (N = 330)^a^				Older persons and informal caregivers (N = 200)^b^				P-value for the interaction between predictorand healthcareprovider (yes/no)^c^
	Unstandardized coefficient B	Confidence interval for B			Unstandardized coefficient B	Confidence intervall for B			
		*Lower limit*	*Upper limit*	*P-value*		*Lower limit*	*Upper limit*	*P-value*	
*Intercept*	10.47	10.14	10.80	0.00**	9.03	8.84	9.22	0.00**	0.00**
*Morbidities*	-0.02	-0.06	0.02	0.28	-0.14	-0.15	-0.12	0.00**	0.00**
*Functional limitations*	-0.05	-0.07	-0.03	0.00**	-0.12	-0.13	-0.11	0.00**	0.00**
*Emotional wellbeing*	-0.05	-0.07	-0.03	0.00**	-0.04	-0.05	-0.03	0.00**	0.24
*Pain experience*	-0.18	-0.28	-0.08	0.00**	-0.04	-0.10	0.02	0.17	0.05*
*Cognitive functioning*	-0.12	-0.21	-0.03	0.01*	-0.13	-0.22	-0.04	0.00*	0.85
*Social functioning*	-0.16	-0.21	-0.11	0.00**	-0.01	-0.02	0.02	0.63	0.00**
*Self-perceived health*	-0.18	-0.26	-0.10	0.00**	-0.16	-0.20	-0.12	0.00**	0.68
*Self-perceived QOL*	-0.28	-0.38	-0.19	0.00**	-0.02	-0.07	0.02	0.29	0.00**

^a^ Dependent variable: GWB scores given by healthcare professionals

^b^ Dependent variable: GWB scores given by older persons and informal caregivers

^c^ Dependent variable: GWB scores given by healthcare professionals, older persons, and informal caregivers

* p≤0.05

**p≤0.001

The components *morbidities* and *functional limitations* had stronger associations with GWB scores given by older persons than with scores given by healthcare professionals: for every morbidity present the GWB score based on the preference weights of older persons and informal caregivers declined with 0.14 points, whereas the GWB score based on the preference weights of healthcare professionals declined with 0.02 points. These numbers were 0.12 versus 0.05 for every functional limitation, respectively. On the other hand, the components *pain experience*, *social functioning*, and *self-perceived QOL* had stronger associations with GWB scores given by healthcare professionals than with scores given by older persons and informal caregivers: when pain increased one point on the Likert scale (no pain, moderate pain, severe pain) the GWB score based on the preference weights of older persons and informal caregivers declined with 0.04 point, whereas the GWB score based on the preference weights of healthcare professionals declined with 0.18 points. These numbers were 0.01 versus 0.16 for social functioning, and 0.02 versus 0.28 for self-perceived QOL, respectively.

### Distribution of the CEP’s

Of the 453 cases described in the vignettes from both vignette studies, the majority (84.7%) had no missing data points for the calculation of TOPICS-CEP scores or HP’s CEP. Consequently, both composite outcome measures were calculated for 384 cases.

**Fig 1:**
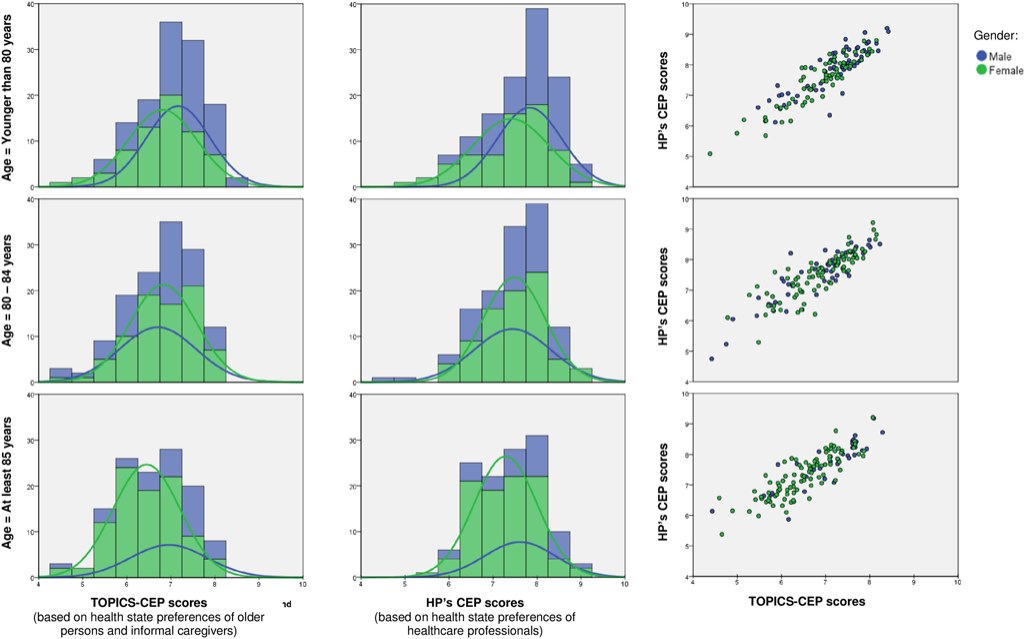
Frequency distribution and correlation matrices for men (blue) and women (green) of HP’s CEP and TOPIC-CEP scores of the case vignettes by age groups (N = 384).

The overall distribution for both measures were tailed to the left (not shown), though became more normalized when stratified by age (Fig. 1). Mean scores (±SD) significantly differed across gender and age groups for both HP’s CEP [(**Men**: 7.695 (0.80); **Women**: 7.43 (0.76); p = 0.001) (**<80**: 7.67 (0.79); **80–84**: 7.50 (0.78); **≥85**: 7.42 (0.76); p = 0.039)] and TOPICS-CEP [(**Men**: 7.01 (0.82); **Women**: 6.73 (0.786; p = 0.001) (**<80**: 7.02 (0.75); **80–84**: 6.83 (0.79); **≥85**: 6.64 (0.80); p = 0.001)].

The minimum and maximum HP’s CEP scores calculated were 4.75 and 9.21, respectively. These scores were 4.38 and 8.42 for TOPICS-CEP. The mean HP’s composite score (±SD) differed from the mean TOPICS-CEP [**HP’s CEP**: 7.53 (0.78); **TOPICS-CEP:** 6.84 (0.79); p<0.001]. The two composite outcome measures were highly correlated (r = 0.88, p<0.001).

**Fig 2:**
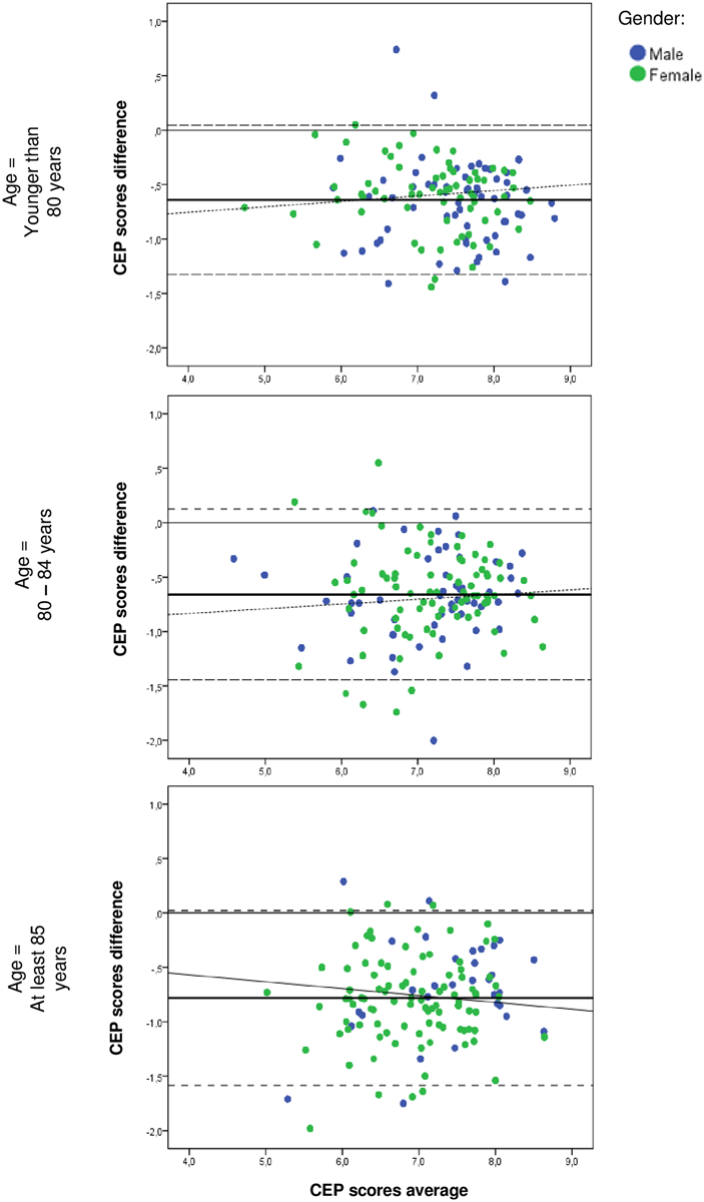
Bland-Altman plots of the variation between HP’s CEP scores and TOPICS-CEP scores against the mean of HP’s CEP and TOPICS-CEP scores per age group (N = 384). Bold solid line represents the average difference between the two scores; broken lines represents the 95% confidence intervals limits.

The Bland-Altman plot showed consistent variability and there were no trends visible across the graphs (Fig. 2). For the cases aged younger than 80 years, the average of HP’s CEP and TOPICS-CEP scores ranged from 4.74 to 8.80 [**80–84:** 4.59 to 8.64; **≥85:** 5.02 to 8.64], the difference between the scores ranged from-1.44 to 0.74 [**80–84:** -2.00 to 0.55; **≥85:** -1.98 to 0.29], the average bias was-0.64 [**80–84:** -0.66; **≥85:** -0.78] and the level of agreements were-1.33 and 0.05 [**80–84:** -1.44 and 0.12; **≥85:** -1.15 and 0.02].

## Discussion

Our primary findings indicate that the weights of TOPICS-CEP’s components based on the health state preferences of healthcare professionals differed significantly from those based on the preferences of older persons and informal caregivers. These findings are in line with other studies exploring the discrepancies between older persons and healthcare professionals concerning health state preferences [15, 16].

Our results indicate that the presence of morbidities and functional limitations in the vignette cases have a greater impact on the GWB scores given by older persons and informal caregivers than on the scores given by healthcare professionals. However, the presence of increased levels of pain experience, hampering of social functioning, and an decrease of self-perceived QoL status have a greater impact on the GWB scores given by healthcare professionals than on the scores given by older persons and informal caregivers. Furthermore, our results suggest that healthcare professionals’ number of *years of experience* influence the preference weight of *morbidities*. The higher the numbers of years of experience the lower the negative impact of the number of morbidities on GWB scores. To explore whether a change of 0.005 point per additional year is clinically relevant further research needs to be conducted.

Finally, our results show that the mean differences between HP’s CEP and TOPICS-CEP scores were not close to zero for any of the age groups, which indicates that the two composite outcome measures are systematically producing different results. Yet, to understand whether these systematic differences are clinically relevant further research needs to be conducted. Moreover, in the Bland-Altman plot there were no trends visible in any of the age groups.

Our results and implications need to be interpreted in light of several limitations. First, the vignettes used in the two studies were not the same. This means that the GWB of a vignette case was never assessed by both an older person or informal caregiver and a healthcare professional. However, all vignettes were based on empirical data derived from the TOPICS-MDS National Database and the cases were all plausible health state combinations. Consequently, none of the raters had to assess impossible health state combinations, e.g. a case that has eight morbidities and experiences severe pain, but does not have any functional limitations. With this in mind, we do not expect that the use of a different set of vignettes influenced our findings.

Second, we compared the components’ preference weights between the various professions and explored the influence of work experience on these weights. However, we have not studied the influence of personal characteristics of the professionals, such as gender and age, on the preference weights. This was a well-considered decision as the aim of our study was to establish a CEP based on the preference weights of a random sample of healthcare professionals.

## Conclusion

If more than one outcome is important for effectiveness evaluation or if an intervention has the potential to improve more than one health domain, a CEP can efficiently deal with the issue of multiplicity, e.g. in elderly care. By using a preference-weighted multifaceted outcome measure, such as TOPICS-CEP, the relative importance of the various outcomes is taken into account. At the macro level, TOPICS-CEP which is based on older persons’ health state preferences may be considered as a general patient reported outcome measure to be used for evaluating healthcare interventions for (frail) older subjects.

When examining the effectiveness of healthcare interventions in elderly care need to consider the discrepancies between the health state preferences of older persons and healthcare professionals. Failure to recognize these discrepancies may lead to incorrect interpretation of the findings and the establishment of inappropriate healthcare policies. Furthermore, healthcare professionals need to keep in mind that their own health state preferences may not be the same as those of their older patients. This provides a good argument for shared decision making in healthcare.

## Supporting Information

S1 Appendix (DOCX)

S2 Appendix (DOCX)
