# Supplementary material for: Comparing the Health State Preferences of Older Persons, Informal Caregivers and Healthcare Professionals: A Vignette Study
Source: PLoS One. 2015 Mar 4;10(3):e0119197. doi: 10.1371/journal.pone.0119197 (PMC4349801; doi:10.1371/journal.pone.0119197)
Supplement: S1 Appendix — (DOCX) [file pone.0119197.s001.docx]

**Appendix 1:** The health outcome domains, vignette items, levels of the items, and aggregated items included in the vignettes from both studies.

| **Outcome**  **Domains** | **Vignette items** | **Outcomes**  **(levels)** | **Descriptives**  **of cases included in first vignette study: older persons and informal caregivers**  **(N, %)** | **Descriptives**  **of cases included in second vignette study: healthcare professionals**  **(N, %)** |
| --- | --- | --- | --- | --- |
| Morbidity  (Local and national health monitor)[[17](#_ENREF_17)] | Presence of:  Dementia; Depression; Incontinence; Stroke, CVA or TIA; Hip fracture; Panic or anxiety disorder; Dizziness with falling; Vision disorder; Asthma; Osteoporosis; Diabetes; Arthritis; Heart failure; Form of cancer; Complaints due to benign enlarged prostate; Fracture other than hip fracture; Hearing disorder | Number of diseases present; *counting the number of health items “Present”*  *Range: 0-17* | Mean: 3.5  SD: 2.0 | Mean: 3.3  SD: 2.3 |
| Functional limitations  (modified KATZ-ADL Index) [[12](#_ENREF_12)] | Needing help with:  Brushing hair; Going to the toilet; Taking medication; Sitting down and getting up from chair; Getting dressed; Travelling; Handling finances; Grocery shopping; Walking about; Taking a bath or shower; Housekeeping; Preparing a meal; Eating; Using the telephone | Number of limitations in (I)ADL; *counting the number of physical functioning items “Help needed”*  *Range: 0-15* | Mean: 3.0  SD: 3.5 | Mean: 4.4  SD:4.2 |
| Emotional wellbeing  (Rand-36, mental health subscale) [[13](#_ENREF_13)] | Feeling down; Feeling blue; Feeling nervous; Feeling happy; Feeling calm | Raw mental health score; *Calculating the raw score of the five mental health items, each ranging from 1 to 6 (Always – Never)*  *Range: 5-30* | Mean:10.4  SD: 4.7 | Mean:9.77  SD: 4.4 |
| Pain experience  (Single item EQ-5D+C) [[18](#_ENREF_18)] | Pain experience | No  Moderate  Severe | 130 (44.7)  127 (43.6)  34 (11.7)  (N=291) | 50 (39.4)  60 (47.2)  17 (13.4)  (N=127) |
| Cognitive functioning  (Single item EQ-5D+C) [[18](#_ENREF_18)] | Cognitive problems | No  Moderate  Severe | 222 (77.1)  64 (22.2)  2 (0.7)  (N=288) | 74 (58.6)  37 (29.1)  16 (12.6)  (N=127) |
| Social functioning  (Single item RAND-36) [[13](#_ENREF_13)] | Social activities hampered by physical health or emotional problems | Never  Rarely  Sometimes  Mostly  Continuous | 217 (75.9)  19 (6.6)  29 (9.1)  6 (2.1)  18 (6.3)  (N=289) | 90 (52.6)  25 (14.6)  22(12.9)  14 (8.2)  20 (11.7)  (N=171) |
| Self-perceived health  (Single item, RAND-36) [[13](#_ENREF_13)] | Self-perceived health in general | Excellent  Very good  Good  Reasonable  Poor | 18 (6.3)  20 (7.0)  127 (44.3)  108 (37.6)  14 (4.8)  (N=287) | 7 (4.0)  9 (5.2)  66 (38.2)  73 (42.2)  18 (10.4)  (N=173) |
| Satisfaction with quality of life (QOL)  (Single item formed using phrasing similar to self-perceived health question, RAND-36) [[13](#_ENREF_13)] | Self-perceived QOL in general | Excellent  Very good  Good  Reasonable  Poor | 26 (9.1)  40 (14.0)  170 (59.4)  41 (14.4)  9 (3.1)  (N=286) | 5 (2.9)  17 (9.8)  94 (54.0)  54 (31.0)  4 (2.3)  (N=174) |
