# Supplementary material for: Comparing the Health State Preferences of Older Persons, Informal Caregivers and Healthcare Professionals: A Vignette Study
Source: PLoS One. 2015 Mar 4;10(3):e0119197. doi: 10.1371/journal.pone.0119197 (PMC4349801; doi:10.1371/journal.pone.0119197)
Supplement: S2 Appendix — (DOCX) [file pone.0119197.s002.docx]

**Appendix 2.**

Example vignette

| **Case description**  Mrs. X is 85 years old. She is a widow and lives independently without others.  **Diseases**  Mrs. X has faced 10 health problems in the past 12 months. These include stroke, asthma COPD, incontinence, arthritis, osteoporosis, fracture other than hip fracture, dizziness with falls, depression, anxiety and problems with vision. She experiences severe pain or other symptoms.  **Cognitive functioning**  Mrs. has some problems with her memory, attention and thought.  **Activities in daily living**  Mrs. requires help with none of seven self-care tasks. This means that she does not need any help with bathing or showering, dressing, walking, rising from a chair, combing her hair, going to the toilet and when eating.  Mrs. requires help with four out of seven more complex tasks. She needs help with travelling, grocery shopping, preparing a meal, and housekeeping. Mrs. does not need any help using the telephone, handling money, and taking her medication.  Mrs. does not use incontinence products.  **Emotional wellbeing**  The past month Mrs. has felt nervous sometimes, calm sometimes, depressed sometimes, and happy very often. She has never felt so sad that nothing could cheer her up.  **Social functioning**  Mrs. X’s social activities, such as visiting family and friends, has been hampered continuously by her physical health or emotional problems the last month.  **Self-perceived health and self-perceived quality of life**  Mrs. X says her health is good and her quality of life is good, meaning she enjoys her life and is satisfied with it. |
| --- |
